# Pharmacogenomic knowledge representation, reasoning and genome-based clinical decision support based on OWL 2 DL ontologies

**DOI:** 10.1186/s12911-015-0130-1

**Published:** 2015-02-22

**Authors:** Matthias Samwald, Jose Antonio Miñarro Giménez, Richard D Boyce, Robert R Freimuth, Klaus-Peter Adlassnig, Michel Dumontier

**Affiliations:** Center for Medical Statistics, Informatics, and Intelligent Systems, Medical University of Vienna, Spitalgasse 23, 1090 Vienna, Austria; Institute of Medical Informatics, Statistics, and Documentation; Medical University of Graz, Auenbruggerplatz 2, 8036 Graz, Austria; Department of Biomedical Informatics, University of Pittsburgh, 5607 Baum Blvd, Suite 419, Pittsburgh, PA 15206-3701 USA; Department of Health Sciences Research; Mayo Clinic, 200 First Street SW, Rochester, MN 55905 USA; Medexter Healthcare GmbH, Borschkegasse 7/5, 1090 Vienna, Austria; Stanford Center for Biomedical Informatics Research, Stanford University, 1265 Welch Road, Stanford, CA 94305-5479 USA

**Keywords:** Pharmacogenomics, Ontology, Automated reasoning, Personalized medicine, Clinical decision support

## Abstract

**Background:**

Every year, hundreds of thousands of patients experience treatment failure or adverse drug reactions (ADRs), many of which could be prevented by pharmacogenomic testing. However, the primary knowledge needed for clinical pharmacogenomics is currently dispersed over disparate data structures and captured in unstructured or semi-structured formalizations. This is a source of potential ambiguity and complexity, making it difficult to create reliable information technology systems for enabling clinical pharmacogenomics.

**Methods:**

We developed Web Ontology Language (OWL) ontologies and automated reasoning methodologies to meet the following goals: 1) provide a simple and concise formalism for representing pharmacogenomic knowledge, 2) finde errors and insufficient definitions in pharmacogenomic knowledge bases, 3) automatically assign alleles and phenotypes to patients, 4) match patients to clinically appropriate pharmacogenomic guidelines and clinical decision support messages and 5) facilitate the detection of inconsistencies and overlaps between pharmacogenomic treatment guidelines from different sources. We evaluated different reasoning systems and test our approach with a large collection of publicly available genetic profiles.

**Results:**

Our methodology proved to be a novel and useful choice for representing, analyzing and using pharmacogenomic data. The Genomic Clinical Decision Support (Genomic CDS) ontology represents 336 SNPs with 707 variants; 665 haplotypes related to 43 genes; 22 rules related to drug-response phenotypes; and 308 clinical decision support rules. OWL reasoning identified CDS rules with overlapping target populations but differing treatment recommendations. Only a modest number of clinical decision support rules were triggered for a collection of 943 public genetic profiles. We found significant performance differences across available OWL reasoners.

**Conclusions:**

The ontology-based framework we developed can be used to represent, organize and reason over the growing wealth of pharmacogenomic knowledge, as well as to identify errors, inconsistencies and insufficient definitions in source data sets or individual patient data. Our study highlights both advantages and potential practical issues with such an ontology-based approach.

**Electronic supplementary material:**

The online version of this article (doi:10.1186/s12911-015-0130-1) contains supplementary material, which is available to authorized users.

## Background

Every year, hundreds of thousands of patients experience treatment failure or adverse drug reactions (ADRs). Treatment response rates in 14 therapeutic areas have varied from 25-80% with many drugs falling in the range of 50-75% [1]. Approximately 2.4 out of every 1000 persons in the US visit the emergency department every year due to an ADR [2]. The efficacy and safety of therapies in the “average patient” have been historically obtained using randomized clinical trials where the variable patient attributes are randomly distributed so as not to confound outcome measurements. Recent advances in genomics have enabled a much better understanding of specific molecular contributions to variability in phenotypic response [3]. Much of this variability can be explained by genetic differences between patients, which can strongly influence how medications are metabolized and the degree to which they interact with biochemical targets [4]–the focus of a discipline called pharmacogenomics [5,6].

**Figure 1:**
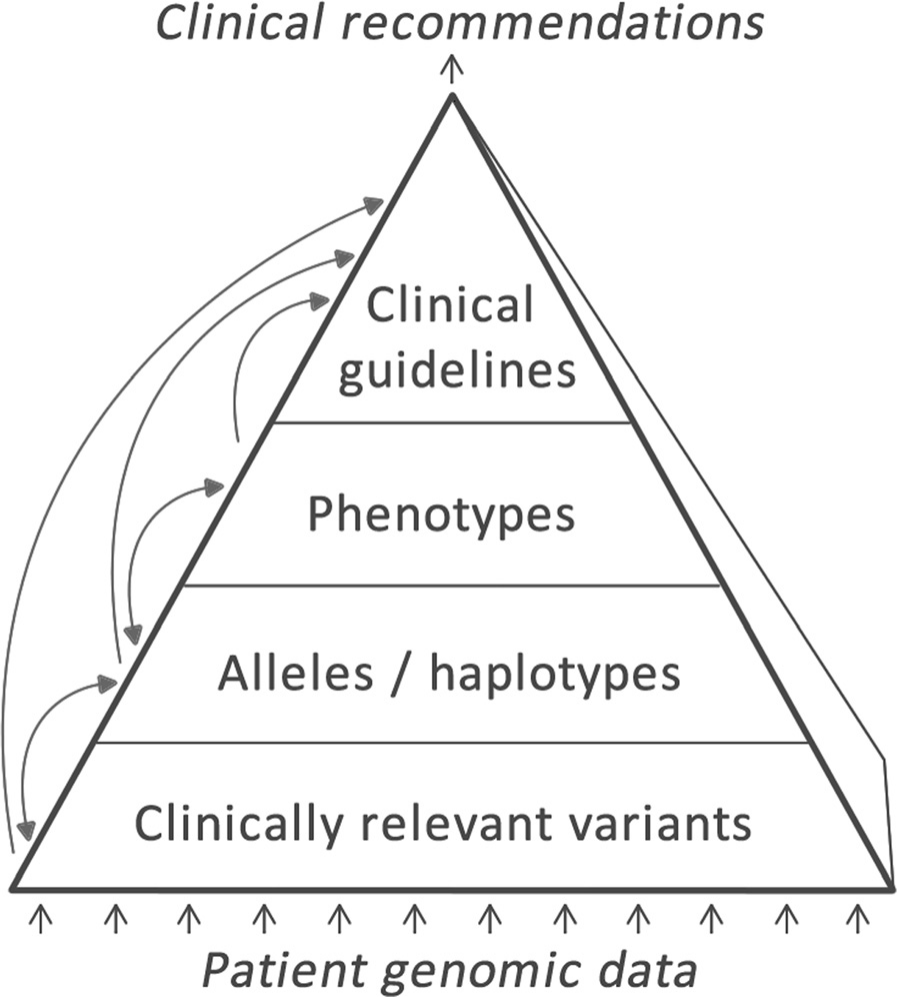
**Overview of types of information and inference in the pharmacogenomics domain.** Raw genetic patient data are at the bottom and clinical recommendations are at the top of the hierarchy. Through a series of logical inference steps, intermediate classifications are generated based on raw data. Inference steps are visualized as arrows.

To make the use of pharmacogenomic biomarkers more clinically effective, the potentially large and complex data yielded by genotyping or sequencing need to be reduced to more manageable, higher-level characteristics such as alleles, haplotypes or phenotypes that can help to predict drug response (Figure 1). Genetic characteristics and higher-level classifications need to be clearly and unambiguously defined in order to avoid errors and inconsistencies in downstream clinical applications. However, the primary knowledge needed for clinical pharmacogenomics is currently captured in either unstructured text or semi-structured formalisms. This makes it difficult to integrate data across relevant sources to enable automated data quality assurance. The lack of formal semantics for the data is a source of potential ambiguity that makes it difficult to create reliable information technology systems for enabling clinical pharmacogenomics.

As alternatives to traditional data warehouses and relational databases, semantic technologies have gained broad acceptance over the past decade as key for addressing problems of biomedical knowledge representation, integration and reasoning [7-10]. In particular, technologies based on the Resource Description Framework (RDF) [11] and the Web Ontology Language (OWL, OWL 2) [12] are especially promising as a logic-based knowledge representation formalism to construct, instantiate and reason with biomedical ontologies. OWL provides a set of standard profiles, each with well-understood expressiveness/tractability tradeoffs [13].

Here we explore the use of ontologies, RDF/OWL and automated reasoning to meet the following goals:

- Providing a simple yet rigorous formalism for representing pharmacogenomic knowledge
- Finding errors and lacking definitions in pharmacogenomic knowledge bases
- Automatically assigning alleles and phenotypes to patients
- Matching patients to clinically appropriate pharmacogenomic guidelines and clinical decision support messages
- Facilitating the detection of inconsistencies and overlaps between pharmacogenomic treatment guidelines from different sources.

In particular, we have developed an OWL 2 ontology that brings raw genetic markers and inferred treatment recommendations within a single, coherent model. This ontology contains a concise logical formalization of clinical pharmacogenomic definitions and rules, forming a knowledge base that can be used as a common platform for different pharmacogenomic assays and decisions support systems. The ontology is used for automated reasoning to detect potential errors in pharmacogenomic definitions, and to automatically infer clinical decision support messages that match a patient’s individual genetic profile. In the following sections, we describe our proof-of-concept implementation of the Genomic Clinical Decision Support (‘Genomic CDS’) ontology.

## Methods

All resources and scripts described in this section are freely available on the web at https://code.google.com/p/genomic-cds/.

### Selection of relevant pharmacogenomic markers

We downloaded (June 2014) and compiled a non-redundant list of 43 genes and 336 Single Nucleotide Polymorphisms (SNPs) relevant to clinical pharmacogenomics and identified by dbSNP [14] identifiers by merging data from: (1) the list of ‘very important pharmacogenes’ and their associated SNPs made available by the Pharmacogenomics Knowledge Base (PharmGKB) [15]; (2) the PharmaADME core gene list [16]; and (3) markers mentioned in FDA drug labels [17], excluding markers of somatic, non-inherited mutations. The majority of the markers consisted of SNPs, but the list also contained a small number of other genetic polymorphisms such as indels (segments of the DNA where nucleic acids were inserted and/or deleted). For reasons of simplicity, we will refer to the collection of markers simply as ‘SNPs’ from here on.

SNPs of the following genes were included in the ontology: *ABCB1, ADRB1, BRCA1, CFTR, COMT, CYP1A2, CYP2A6, CYP2B6, CYP2C19, CYP2C9, CYP2D6, CYP3A4, CYP3A5, DPYD, DPYD-AS1, F5, G6PD, HLA-A, HLA-B, HMGCR, IFNL4, LOC100286922, LOC101927831, MED12L, MIR4761, NBR1, P2RY12, RP11-242D8.1, SLCO1B1, SULT1A1, TPMT, UGT1A1, UGT1A10, UGT1A3, UGT1A4, UGT1A5, UGT1A6, UGT1A7, UGT1A8, UGT1A9, VKORC1, ZMIZ1-AS1* and *ZSCAN25*. These genes are pharmacogenes associated with the selected SNPs, as well as–to a small extent–genes with other functions that overlap with the selected SNPs.

### Ontology development

Top-level classes of the ontology were created manually with the Protégé 4 ontology editor [18]. Lower-level classes were automatically created through scripts as described below.

**Figure 2:**
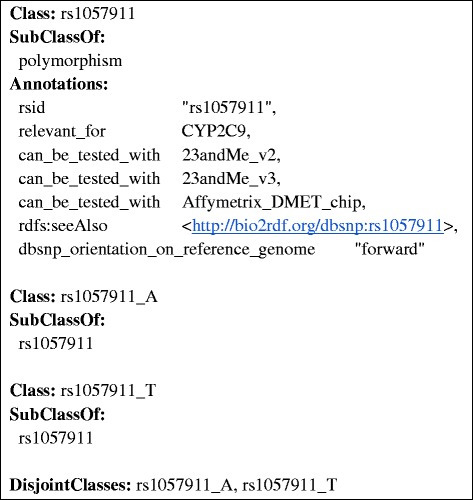
**Example of the representation of the “rs1057911” SNP and its alleles in the ontology.** SNPs are represented as subclasses of “polymorphism”. The “rsid” annotation indicates the dbSNP identifier. The “relevant_for” annotation links SNPs to associated genes. The “can_be_tested_with” annotation associates genetic assays with the SNPs they test for. The “dbsnp_orientation_on_reference_genome” annotation represents the orientation of the SNP provided by the dbSNP repository and is used to match the reference genome orientation when parsing the results of a genomic test. The “rdfs:seeAlso” relation provides a Linked Data representation of the SNP provided by the Bio2RDF project. Alleles are represented as sub-types of the SNP, differing by the specific genetic variation they instantiate.

We used the dbSNP batch query interface to download the dbSNP records for all of the 336 genetic markers. The dbSNP entries were converted to OWL axioms. Information on coverage of specific polymorphisms by different genetic testing panels was extracted from manufacturer data sheets and added to the OWL descriptions. Figure 2 exemplifies how these SNP data were represented in the ontology. A key aspect of the representation is that SNP variants are represented as subclasses of the SNP, and can be used in constructing haplotype expressions with specific SNP variants.

**Figure 3:**
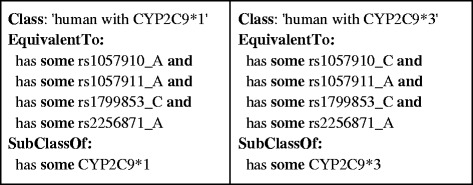
A subset of OWL axioms defining a human with haplotypes CYP2C9*1 and CYP2C9*3 based on alleles in Table **1** **.** The “EquivalentTo” expression indicates the necessary and sufficient conditions to identify a human related to such haplotypes and the “SubClassOf” expression provides the necessary conditions to identify a human with the haplotypes. This representation is optimized for the inference of matching haplotypes from raw SNP data, which is one of the major use-cases of the ontology.

**Figure 4:**
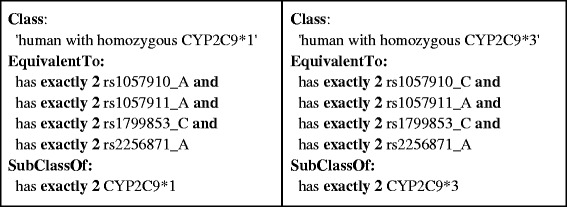
The subset of OWL axioms defining a human with homozygous haplotypes CYP2C9*1 and CYP2C9*3 based on the allele definitions in Table **1** **.** In the “EquivalentTo” expression the OWL axioms indicate the requirement of two copies of every SNP allele to identify a human with a homozygous haplotype, whereas in the “SubClassOf” expression the class is associated with two copies of the haplotype. Consequently, an individual that meets the “EquivalentTo” conditions is also associated to the corresponding homozygous haplotype.

**Figure 5:**
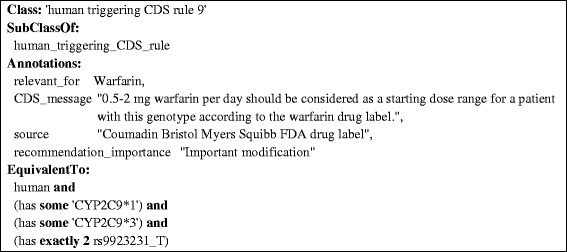
**An excerpt of a CDS rule derived from the warfarin drug label.** This rule provides a specific warfarin dosage range for treatment initiation in patients having CYP2C9 alleles *1 and *3 and being homozygous for the ‘T’ variant of the SNP rs9923231 in the VKORC1 gene. The “relevant_for” annotation indicates the type of drug that this recommendation is related to. The “CDS_message” annotation represents the textual description of the drug dosage recommendation. The “source” annotation provides the name of the source repository where the drug dosage recommendation was available. The “recommendations_importance” is a manually curated annotation indicates whether a rule recommends standard treatment, a minor deviation from standard treatment, or an important/critical modification of treatment.

**Figure 6:**
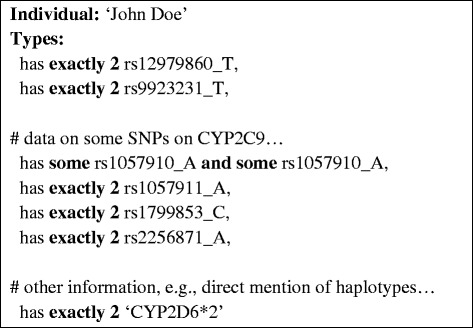
**An example of how pharmacogenomic findings about an individual patient - such as heterozygous and homozygous SNP and allele variants-can be represented.**

Haplotype definitions were retrieved from the Pharmacogenomics Knowledge Base (PharmGKB) [15] and were further curated manually to fit them into a uniform table format. We created a PHP script to parse PharmGKB haplotype/allele tables in order to create OWL axioms representing the definitions in these tables. Since haplotypes are defined by sets of SNP variants, we formalized these as ‘necessary and sufficient’ conditions expressed as equivalentClass axioms. We used qualified cardinality restrictions to assert whether the haploytype consisted of one or two alleles, for heterozygous and homozygous scenarios respectively. We then used an OWL reasoner to check the logical consistency of the ontology. The reasoner accomplished this by inferring the logical consequences of the definitions, axioms, and facts that made up the ontology and that were generated from the haplotype/allele tables in PharmGKB. Example haplotype/allele information is shown in Table 1 along with corresponding OWL axioms in Figures 3 and 4. Figures 5 and 6 exemplify how these haplotypes/alleles are used for representing decision support rules or individual patient data.

**Table 1:** **An excerpt of a translational allele/haplotype table for CYP2C9 taken from PharmGKB**

**Haplotype**	**rs1057910**	**rs1057911**	**rs1799853**	**rs2256871**
CYP2C9*1	A	A	C	A
CYP2C9*3	C	A	C	A

Pharmacogenomic decision support rules and drug response phenotype inference rules were curated from clinical guidelines of the Clinical Pharmacogenetics Implementation Consortium (CPIC) [19] and the Dutch Pharmacogenomics Working Group [20], as well as drug labels approved by the U.S. Food and Drug Administration (FDA). We curated 308 rules providing dosage recommendations covering 65 drugs and 22 rules for inferring drug response phenotypes. Figure 5 shows an OWL representation of a dosage recommendation for the drug warfarin obtained from an FDA product label (see the ‘CDS_message’ annotation).

The final Genomic CDS ontology was created by merging all of the OWL axioms generated in the process described so far.

For reasoning with patient data, individual patients and their genetic profiles were represented as OWL Individuals as exemplified in Figure 6. We created services for automatically creating and classifying/realising such patient representations based on three possible input formats: 23andMe files [21], Variant Call Format (VCF) files [22], and two-dimensional barcodes representing pharmacogenomic data as Medicine Safety Codes [23]. We obtained publicly available patient data in the form of genetic 23andMe V3 profiles from openSNP.org [24], manually altering some of the SNP data in order to create a unique, virtual profile, and converting the SNP data into OWL axioms describing the genetic profile of a virtual patient.

Figures 2, 3, 4, 5 and 6 provide an example of the major reasoning tasks necessary to infer matching CDS recommendations from raw data with the ontology. The reasoning process can be outlined as follows: Patient data generated from a pharmacogenomic assay is converted to OWL axioms (i.e., an OWL individual) and entered into the system (Figure 6). The patient data contains information about some SNPs associated with the CYP2C9 gene, which the reasoner can match with the allele/haplotype definitions for this gene (Figures 4 and 5). The reasoner infers from the SNP pattern that the patient is heterozygous for this gene, and that the patterns matches a combination of CYP2C9*1 and CYP2C9*3. The reasoner then matches the raw data plus the newly inferred data with the clinical decision support rules, finding a match with a rule for warfarin dosing (Figure 5).In this case, the OWL Individual is now associated with the OWL class of the rule and the corresponding decision support message: “0.5-2 mg warfarin per day should be considered as a starting dose range for a patient with this genotype according to the warfarin drug label”. This decision support message would be combined withmessages generated for other medications and then be displayed by the system in order to guide the treatment of the patient.

### OWL reasoner evaluation

The Genomic CDS ontology makes extensive use of qualified cardinality restrictions greater than one (e.g., “human and has exactly 2 CYP2C19*1”) and therefore does not fit into one of the restricted OWL 2 profiles such as OWL 2 EL, QL or RL. We therefore focused on the evaluation of reasoners supporting the computationally challenging OWL 2 DL profile [13].

As a testing environment we used a machine with Windows 7 Professional, Java version 1.6.0_29-b11 and 64 bit platform running on an Intel Core i5-2430 M and 4GB of memory. We developed a testing application with OWLAPI 3.4.3 and the following OWL 2 reasoners: TrOWL 1.3 [25], HermiT 1.3.8 [26], the Pellet 2.3.1 [27], and Fact++ 1.6.2 [28].

In the evaluation, the system had to perform all major functionalities of OWL reasoning in a single run: 1) consistency checking to ensure that the ontology is consistent (otherwise inferences could be invalid), 2) inferring the class hierarchy, 3) realizing the ontology, i.e., associating the patient–represented as an OWL individual–with matching OWL classes and inferring properties.

### Bulk-processing of publicly available genetic profiles

We tested the ontology-based reasoning with publicly available human genotype datasets to evaluate its potential for automated genome-based clinical decision support. Human genotype datasets generated by the 23andMe and VCF assay–containing data on almost a million polymorphisms per patient–were collected from the openSNP repository, which collects genetic test results that are in the public domain. In total, 935 genetic profiles were processed and inferred recommendations were obtained.

To generate the recommendations for all genotype files, we developed a custom application for automatically loading genetic profiles in sequence, populating our ontology with each genetic profile, inferring alleles and clinical decision support rules and finally calculating population statistics.

## Results and discussion

Our methodology proved to be a novel and useful choice for representing, analyzing and using pharmacogenomic data. The ontology files are contained in Additional file 1 and detailed statistics about reasoning results are contained in Additional file 2. The final ontology represented 336 SNPs with 707 variants; 665 haplotypes related to 43 genes; 22 rules related drug-response phenotypes; and 308 clinical decision support rules.

### Analysis and improvement of allele definitions and decision support rules

In generating the ontology from haplotype definition tables, we identified some sources of problems in the PharmGKB tables we used which were caused by poor haplotype definitions and which led to inconsistencies in the ontology during early stages of ontology development. A major problem was that several alleles were underspecified, i.e., the SNPs listed for one haplotype overlapped with all the SNPs of another haplotype, making the haplotypes indistinguishable by the data in the table alone. Another problem was that some of the SNP entries used in the PharmGKB tables had been deprecated in the current release of dbSNP.

OWL reasoning helped identify some CDS rules with overlapping target populations but differing treatment recommendations. For example, the reasoner highlighted an overlap in the patient populations targeted by two treatment recommendations for azathioprine issued by CPIC and the Dutch Pharmacogenomics Working Group, which made one patient population a subset of the other patient population (Table 2). This is not an error in the data–such discrepancies between guidelines from different groups are to be expected. Our system can make such cases better manageable by inferring overlaps and differences between guidelines and reporting different classifications/recommendations. In total, 57 out of the 330 phenotype of decision support rules in the ontology were targeting equivalent patient populations or patient populations that were subgroups of other patient populations (i.e., a patient meeting criteria of one rule was a subclass of a patient meeting criteria of another rule, potentially with conflicting clinical recommendations).

**Table 2:** **Patients triggering rule 27 (issued by CPIC) are inferred by the reasoner to be a subclass of patients triggering rule 35 (issued by the Dutch Pharmacogenomics working group, both rules concern the substance azathioprine)**

**ID**	**Curated OWL 2 axiom**	**Recommendation**
27	Has some TPMT*1 and has some (TPMT*2 or TPMT*3A or TPMT*3B or TPMT*3C or TPMT*4)	If disease treatment normally starts at the “full dose”, consider starting at 30-70% of target dose (e.g., 1–1.5 mg/kg/d), and titrate based on tolerance. Allow 2–4 weeks to reach steady state after each dose adjustment.
35	Has some TPMT*1 and has some (TPMT*2 or TPMT*3 or TPMT*4 or TPMT*5 or TPMT*6 or TPMT*7 or TPMT*8 or TPMT*9 or TPMT*10 or TPMT*11 or TPMT*12 or TPMT*13 or TPMT*14 or TPMT*15 or TPMT*16 or TPMT*17 or TPMT*18)	Select alternative drug or reduce dose by 50%. Increase dose in response of hematologic monitoring and efficacy.

This means that every patient who meets criteria for the recommendations given under rule 35 also meets the criteria for recommendations given under rule 27. This inference is based on the highlighted statements in this table. The knowledge that TPMT*3A, *3B and *3C alleles are subclasses of TPMT*3 is captured in the ontology.

### Inferences made for publicly available genotypes

We took a cautious approach in defining ‘necessary and sufficient’ conditions for assigning alleles. Specifically, we tried to take SNPs of rare variants (i.e., with very low minor allele frequency) into account in order to minimize the likelihood of incorrectly inferring common haplotypes. Some publicly available genetic profiles did not include data on these SNPs so that haplotype inference rules were not triggered. This reduced the number of inferences made over the collection of SNPs.

Because of the strict criteria we used, only a modest number of clinical decision support rules were triggered for the collection of 943 public genetic profiles: An average of 188 SNPs in each genetic profile matched one of the polymorphisms in the ontology, and nearly 6 decision support rules were, on average, triggered by each profile. Detailed data on haplotype inferences are listed in Additional file 2.

### Significant performance differences among OWL 2 reasoners

We evaluated the performance of a variety of OWL 2 DL reasoners to reason with the CDS ontology and data of individual genetic profiles. We found significant performance differences between TrOWL and other openly available OWL2 DL reasoners (Table 3). TrOWL returned results within a few seconds, while other reasoners failed to provide results within 3 hours.

**Table 3:** **Time taken by different reasoners for classifying and realising the demo ontology**

**Reasoner**	**Median time required for classification**
TrOWL	**4.62 seconds** (five runs: 4.71 s; 4.62 s; 4.57 s; 4.66 s; 4.58 s)
HermiT	Did not terminate within 3 hours
Pellet	Did not terminate within 3 hours
Fact++	Repeatedly crashed while loading ontology

TrOWL offers tractable support for OWL 2 by using quality-guaranteed language transformations. In particular, TrOWL utilizes a syntactic approximation of OWL 2 DL in OWL 2 EL for TBox and ABox reasoning. Through this syntactic approximation, TrOWL can have vast performance advantages over other OWL 2 DL reasoners for certain ontologies [29]. Completeness of results may be a concern because of the approach taken by TrOWL, though the system appears to provide complete results for most ontologies [30]. TrOWL produced complete results when tested with smaller or simplified versions of the Genomic CDS ontology (using the HermiT reasoner as a comparison), but there is the possibility that some allele assignments did not occur as expected. For instance, during the early stages of developing the CDS ontology, TrOWL did not report certain classes as unsatisfiable that were correctly reported as unsatisfiable by HermiT. We have shared our finding with the TrOWL team and some of our feedback was taken into account in the development of the reasoner system. Recent versions of TrOWL are expected to produce complete inferences with our ontology while retaining the performance advantage of TrOWL over other reasoners (Yuan Ren, personal communication). This makes TrOWL the preferred solution for reasoning with the Genomic CDS ontology or similarly structured ontologies.

Reasoning performance is frequently an issue with potentially large and/or complex biological datasets. Approaches towards reducing the complexity of biomedical ontologies have been proposed. For example, Hoehndorf *et al.* presented a methodology for a lossy transformation of OWL ontologies so they adhere to the performance-optimized OWL 2 EL profile [31].

Only a modest number of decision support rules were triggered in our evaluation. This is because many of the publicly available genetic profiles were lacking information on SNPs which were necessary for calling certain alleles/haplotypes. Our work highlights a general issue of rules used for specifying alleles/haplotypes, which could be seen as a trade-off between precision and recall. In this context, optimizing for precision means avoiding erroneously calling common alleles by including very rare SNPs in the definitions. Unfortunatly, the SNPS might not be present in the overwhelming majority of patients. Such an approach also has the drawback that currently available, array-based genetic test might not be able to produce results covering all these SNPs. Optimizing for recall, on the other hand, means accepting that the system might not be able to provide recommendations specific to rare variants because only common genetic assays would be used. However, the system would likely be able to infer alleles and phenotypes for a greater proportion of patients.

We also found that there is a lack of clearly defined phenotypes for many pharmacogenes. Grouping alleles/haplotypes into clinically distinct, clearly defined and widely accepted phenotypes would ease the use of genetic biomarkers in clinical practice and clinical trials. We found that drug response phenotypes were mentioned in clinical guidelines, but the descriptions of these phenotypes differed among guideline sources (e.g., a ‘CYP2C19 ultrarapid metabolizer phenotype’ was associated with different CYP2C19 alleles in guidelines from CPIC and the Dutch Pharmacogenomics Working Group, respectively).

### Related work

The Clinical Bioinformatics Ontology (CBO) contains some information about pharmacogenetic variants [32], but does not contain logical axioms for inference of alleles and decision support messages through OWL reasoning. It also has some ontological problems (e.g., class-subclass relations are not used in a consistent manner, the true path rule is not observed), and it currently appears to be unsupported.

The SNP-Ontology [33] was among the first ontological resources aimed at representing genetic variation using the OWL 1 description logic. A variant was defined in terms of the reference sequence, the reference sequence type, the sequence position, and the observed variation. The Suggested Ontology for Pharmacogenomics (SO-PHARM) [34] included the SNP-Ontology along with other ontologies in the Open Biomedical Ontology family in order to provide formalized descriptions of patients pharmacogenomic profiles. These ontologies were formalized in OWL 1 and are therefore unable to conveniently represent relevant knowledge captured in the Genomic CDS ontology, because qualified cardinality restrictions were only introduced with OWL 2. Unfortunately, as indicated by BioPortal records, the SNP-Ontology and SO-PHARM have not been maintained for several years.

GENO [35] is an ontological model of genotype information that aims to support data integration across model organism databases. The goal of GENO is to provide a basic set of classes and predicates sufficient to represent the full range of genotype information using OWL. However, the ontology is in an early stage of development and currently does not represent important pharmacogenomic variants. GENO cannot be used for the kind of reasoning and decision support enabled by the Genomic CDS ontology.

The Variation Ontology (VariO) [36] aims to provide a framework for the description of effects, consequences and mechanisms of variations. VariO is a position specific ontology that can be used to describe effects of variations on DNA, RNA or protein level. VariO itself does not describe actual variation on nucleotide/protein level and does not contain any clinical information. We are investigating possibilities for mapping the Genomic CDS ontology to VariO to facilitate mapping to external genomic databases. Finally, the freely accessible wiki SNPedia [37] provides a light-weight formalism for the logical definition of SNP combinations [38]. While being very handy for simple use-cases, concise definitions can become very long (e.g., when covering several different alleles by enumerating their tag SNPs), because the formalism offers no means for defining intermediary classifications (such as haplotypes as and intermediate between raw SNPs and associated phenotypes). This makes the definitions captured in SNPedia difficult to maintain in light of new haplotype definitions and phenotype inference rules.

The Genomic CDS ontology presented in this work goes beyond the state-of-the-art by providing a coherent, ontology-based framework that is optimized for implementing real-world clinical decision support in pharmacogenomics, as well as a data extraction-transformation, curation and consistency-checking workflow that allows for sustainable long-term maintenance of the knowledge base. We have demonstrated the implementation of our ontology-based methodology in a web service for providing pharmacogenomic clinical decision support [39].

### Limitations and future work

We are currently making progress in integrating Genomic CDS and OWL reasoning into a clinical decision support application, which offers clinical recommendations based on patient data.

We plan to broaden our collaboration with key organizations in the field–such as PharmGKB, the Human Cytochrome P450 (CYP) Allele Nomenclature Committee [40], CPIC or the Dutch Pharmacogenomics working group–to work towards well-defined, formalized and logically coherent representations of alleles, phenotypes and criteria for decision support algorithms. We will also evaluate if the ontology-based formalism we developed could be used as a shared representation for knowledge integration among these organizations.

While the ontology is currently mapped to Bio2RDF resources, it has not yet been mapped to other biomedical ontologies or foundational ontologies. The current release of the ontology does not offer means to model the treatment regime of patients, e.g., which drugs an individual patient is actually being prescribed, what dosages are prescribed or other clinical parameters. Instead, the ontology can be used to generate matching pharmacogenomic treatment recommendations for all drugs in the knowledge base, and further filtering or refinement to create targeted clinical decision support messages needs to be done in external applications. In future work, we will explore the possibility of modelling these additional aspects of pharmacotherapy in OWL by extending the ontology and/or linking to other ontologies for this knowledge domain, such as those developed by Grando *et al.* [41].

The design patterns we used for the Genomic CDS ontology could potentially be applied to other, similar reasoning problems in the area of genetics and personalized medicine, with datasets that are far larger than in the use-case presented here. We will investigate how our methodology could scale to meet such demands.

While the framework described in this paper facilitates data representation and reasoning, several barriers to the clinical implementation of pharmacogenomic decision support remain. Processes need to be defined and implemented to make pharmacogenomic data available for broad patient populations, and practical models for integrating decision support into existing clinical workflows need to be found. These barriers are difficult to tackle in light of the heterogeneity of healthcare systems across different regions. We are currently working on a system that allows pharmacogenomic data to be captured in two-dimensional barcodes and to be interpreted with mobile devices [39]. Furthermore, we plan to work on developing best practices for integrating pharmacogenomic data into existing electronic health record infrastructures.

## Conclusions

We described a proof-of-concept semantic reasoning system for pharmacogenomics knowledge representation focused on clinical decision support. It needs to be emphasized that the goal of the work presented here was to analyze if and how these technologies can be applied, rather than attempting to unequivocally define the variants required for specifying alleles or phenotypes–a goal that can only be accomplished through ongoing work in the international pharmacogenomics research community.

The ontology-based framework we developed can be used to represent, organize and reason over the growing wealth of pharmacogenomic knowledge, as well as to identify errors, inconsistencies and lacking definitions in source data sets or individual patient data. It can be applied both in pre-clinical scenarios (e.g., as a reference taxonomy for pharmacogenomic research), as well as clinical applications (pharmacogenomic decision support, patient stratification in clinical trials). Since it leverages OWL 2 and RDF technologies, it can be easily connected to a vast collection of biomedical information resources, and used with a wide variety of tools.

### Availability of supporting data

The data sets supporting the results of this article are included within the article and its additional files.

## Additional files

Additional file 1:
**Ontology files.**
Additional file 2:
**Inference results and statistics.**
